# *Mycobacterium tuberculosis* lineages and anti-tuberculosis drug resistance in reference hospitals across Viet Nam

**DOI:** 10.1186/s12866-016-0784-6

**Published:** 2016-07-28

**Authors:** Van Anh Thi Nguyen, Anne-Laure Bañuls, Thanh Hoa Thi Tran, Kim Lien Thi Pham, Thai Son Nguyen, Hung Van Nguyen, Ngoc Lan Thi Nguyen, Nam Lien Thi Nguyen, Duc Anh Dang, Guy B. Marks, Marc Choisy

**Affiliations:** 1 Department of Bacteriology, National Institute of Hygiene Epidemiology, Hanoi, Vietnam; 2 MIVEGEC (IRD 224-CNRS 5290-Université de Montpellier), Centre IRD, Montpellier, France; 3 Department of Microbiology, Hospital 103, Military Medical University, Hanoi, Vietnam; 4 Department of Microbiology, National Lung Hospital, Hanoi, Vietnam; 5 Department of Microbiology, Pham Ngoc Thach Hospital, Ho Chi Minh city, Vietnam; 6 Department of Microbiology, Hue Central Hospital, Hue, Vietnam; 7 Woolcock Institute of Medical Research, University of Sydney, Sydney, Australia; 8 South Western Sydney Clinical School, UNSW, Sydney, Australia; 9 Oxford University Clinical Research Unit, Hanoi, Vietnam; 10 Laboratory of Tuberculosis, Department of Bacteriology, National Institute of Hygiene and Epidemiology, Hanoi, 10000 Vietnam

**Keywords:** Tuberculosis, *Mycobacterium tuberculosis*, Lineage, Drug resistance, Reference hospital, Vietnam

## Abstract

**Background:**

*Mycobacterium tuberculosis*, the tuberculosis (TB) pathogen, despite a low level of genetic diversity, has revealed a high variety of biological and epidemiological characteristics linked to their lineages, such as transmissibility, fitness and propensity to acquire drug resistance. This has important implications for the epidemiology of TB. We conducted this first countrywide cross-sectional study to identify the prevalent *M. tuberculosis* lineages and to assess their epidemiological associations and their relation to drug resistance. The study was conducted among isolates acquired in reference hospitals across Vietnam. Isolates with drug susceptibility testing profiles were identified for their lineages by spoligotyping. Logistic regression was used to investigate the association of *M. tuberculosis* lineages with location, age and sex of the patients and drug resistance levels.

**Results:**

Results showed that the most prevalent lineage was Beijing (55.4 %), followed by EAI (27.5 %), T (6.4 %), LAM (1.3 %), Haarlem (1 %) and Zero type (0.3 %). The proportion of Beijing isolates in the North (70.4 %) and the South (68 %) was higher than in the Centre (28 %) (OR = 1.7 [95 % CI: 1.4–2.0], *p* < 0.0001), whereas the proportion of EAI isolates in the North (7.1 %) and the South (17 %) was much lower compared with the Centre (59 %) (OR = 0.5 [95 % CI: 0.4–0.6], *p* < 0.0001). Overall, Beijing isolates were the most likely to be drug-resistant and EAI isolates were the least likely to be drug-resistant, except in the South of Vietnam where EAI is also highly drug-resistant. The proportion of Beijing isolates was significantly higher (*p* < 0.01), and the proportion of EAI isolates was significantly lower (*p* < 0.05) in younger patients. The proportion of drug-resistance was higher in isolates collected from male patients and from patients in the middle age groups.

**Conclusions:**

The findings suggest ongoing replacement of EAI lineage, which is mainly more drug-susceptible with highly drug-resistant Beijing lineage in all studied regions of Vietnam. Male patients of working ages should be the focus for better control to prevent the emergence of drug-resistant TB.

**Electronic supplementary material:**

The online version of this article (doi:10.1186/s12866-016-0784-6) contains supplementary material, which is available to authorized users.

## Background

Drug resistant tuberculosis (TB) is one of the main challenges for TB control worldwide due to the emergence of multi-drug resistant (MDR) *Mycobacterium tuberculosis* [1]. Globally, during the period 1994 to 2010, 3.4 % of new and 19.8 % of retreated TB cases was estimated to be MDR-TB (resistant to isoniazid and rifampicin, the two most potent first-line drugs) [2]. In 2014, an estimated 480,000 people developed MDR-TB. By 2015, 105 countries had reported cases of extensively drug-resistant TB (XDR-TB) defined as MDR-TB plus resistance to at least a fluoroquinolone and an injectable second-line drug. This problem, among others, has led to a reduction in treatment success rate [3].

Extensive molecular studies of *M. tuberculosis*, the TB pathogen, have revealed a very low level of genetic diversity [4]. Nevertheless, the genotypic variation, especially the bacterial lineages, is associated with variation in transmission capacity and in the propensity to acquire drug resistance [5–9]. This means that regional variation in the prevalence of specific lineages or sub-lineages can have consequences for the observed epidemiology of TB. Studies showed that Beijing genotypes have been emerging in Vietnam and elsewhere [10–13]. These genotypes are known to be associated with high prevalence of drug resistance [12, 14–16] and high rates of disease transmission [10]. It is feared that these genotypes will spread further in the coming years and influence the development of the TB epidemic [7, 11, 13, 14, 16–20].

In Vietnam, the National TB Control Program has reported that the prevalence of TB was higher in the South of the country (256/100 000), than in the North (162/100 000) and the Center (152/100 000) [21]. Our previous study found that Beijing lineage was the most prevalent in Northern Vietnam, especially in urban areas [10]. Based on genotyping and epidemiological traits, we suggested that strains of this lineage have been imported and spread within urban areas and from urban to rural areas. This spread of Beijing is potentially a major concern, because among other reasons, it may cause the increase of drug-resistant TB. The second most prevalent lineage was EAI lineage, and more particularly EAI4-VNM sub-lineage, which is specific to Vietnam [10, 14]. This sub-lineage was found more commonly in isolates taken from patients in rural, compared with urban, areas [10]. These findings imply that the genetic spectrum of *M. tuberculosis* isolates in Vietnam is not only distinct from that reported in other nations, but also varies within the country.

TB reference hospitals are at the highest level of TB care in Vietnam, where patients from lower levels of the health-care system are referred for management of treatment failures or recurrent TB. As a consequence of this role, these hospitals have a high concentration of patients with drug-resistant TB. We conducted this cross-sectional study in isolates acquired from these hospitals in three main regions to investigate the prevalence of *M. tuberculosis* lineages and their relation to drug resistance in reference hospitals across Vietnam. This molecular epidemiological study is the first investigation of the association between *M. tuberculosis* lineages/sub-lineages and drug resistance at a country level in Vietnam.

## Methods

### Sample collection

**Fig. 1:**
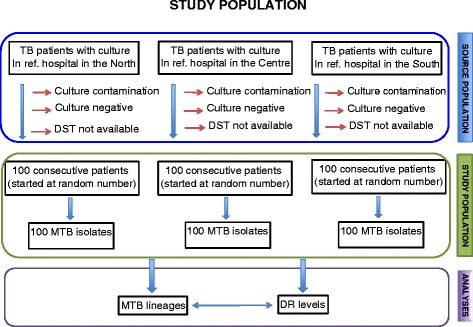
Study population. *Note:* ref.: reference, MTB: *M. tuberculosis,* DR: drug resistance

We acquired 300 *M. tuberculosis* isolates for which drug susceptibility testing (DST) profiles for the four first-line drugs rifampicin (RMP), isoniazid (INH), streptomycin (SM) and ethambutol (EMB) were available, from 3 TB reference hospitals in North (National Lung Hospital), South (Pham Ngoc Thach Hospital) and Centre (Hue Central Hospital) of Vietnam (100 isolates per hospital). The isolates had been collected from patients with treatment failure or recurrent TB during the period 2008 to 2009. In each hospital, the first isolate was selected randomly and the next 99 samples were then collected from consecutive patients (see study population in Fig. 1). During the study period, the TB reference laboratories of the National Lung Hospital and Pham Ngoc Thach Hospital were accredited by the World Health Organization through an External Quality Assurance program implemented by the regional supranational laboratory based in Adelaide, Australia. These laboratories obtained ISO 15189:2007 accreditation in 2010. Samples collected for the study from Hue Central Hospital were sent to the TB reference laboratory at the National Lung Hospital for DST.

### *M. tuberculosis* lineage identification

All isolates, including the control strains *M. tuberculosis* H37Rv and *M. bovis* BCG P3, were typed by spoligotyping with a 43-spacer-membrane as previously described [22, 23]. The results were entered into the international database “SITVITWEB” *(**http://www.pasteur-guadeloupe.fr:8081/SITVIT_ONLINE/**)* [24], in order to identify spoligotypes and phylogenetic lineages. Lineage/sub-lineage of isolates for which the spoligotypes not found in the SITVITWEB database was defined using SPOTCLUST *(**http://tbinsight.cs.rpi.edu/run_spotclust.html**)* [25], and revised by MIRU-VNTR*plus (**http://www.miru-vntrplus.org/MIRU/index.faces**)* [26].

### Data analyses

Logistic regression was used to investigate the association of *M. tuberculosis* lineages with location, age and sex of the patients and drug resistance levels. Each of these variables was considered as a potential confounder for the main effect of the other variables. Interactions between variables were tested when biologically meaningful. Polynomial regressions were tested in order to consider potential non-linear relationships in the logit function. All the statistical analyses were performed in R (R Development Core Team, 2010) and SAS version 9.3 (SAS Institute, Cary, NC).

## Results

### Age and sex distribution of patients whose isolates were included

**Fig. 2:**
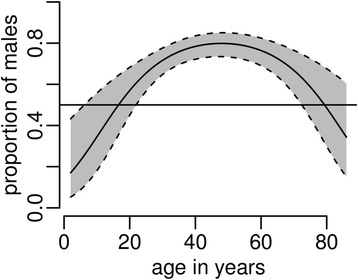
The proportion of male patients by age correcting for location and *M.tuberculosis* lineage. *Note:* The line is the logistic model prediction together with 95 % CI (shaded area)

After adjusting for region and *M. tuberculosis* lineage, it was apparent that the male-to-female ratio differed with age. The proportion of males was higher in patients of working age (25 to 64 years) and lower in the younger and older patients (*p* < 0.001, Fig. 2). The overall male-to-female ratio was 2.4 (71 % vs. 29 %).

### *M. tuberculosis* lineages at the regional reference hospitals

Two isolates showed no band on spoligotyping even after repeated experiments. Among the other 298 isolates, we identified 7 main lineages with 13 sub-lineages (Table 1). Beijing was the most prevalent lineage (165 isolates, 55.4 %). The second most prevalent lineage was EAI (82 isolates, 27.5 %). Only 27 isolates (9.0 %) were of other lineages, including T (19 isolates, 6.4 %), LAM (4 isolates, 1.3 %), Haarlem (3 isolates, 1 %) and Zero (1 isolate, 0.3 %). We found 24 isolates (8.0 %) of undesignated spoligotypes (U) (their lineage could not be identified) (Table 1 and Additional file 1).

**Table 1:** *M. tuberculosis* lineages and sub-lineages at the regional reference hospitals

Lineage	Sub-lineage	Location			
		Total	North	Centre	South
		n (col. %)	n (col. %)	n (col. %)	n (col. %)
Beijing		**165 (55.4)**	**69 (70.4)**	**28 (28.0)**	**68 (68.0)**
EAI		**82 (27.5)**	**7 (7.1)**	**58 (58.0)**	**17 (17.0)**
	EAI2	2 (0.7)		1 (1.0)	1 (1.0)
	EAI2_Manilla	1 (0.3)			1 (1.0)
	EAI4_VNM	44 (14.8)	6 (6.1)	27 (27.0)	11 (11.0)
	EAI5	35 (11.7)	1 (1.0)	30 (30.0)	4 (4.0)
T		**19 (6.4)**	**11 (11.2)**	**4 (4.0)**	**4 (4.0)**
	T1	14 (4.7)	9 (9.2)	2 (2.0)	3 (3.0)
	T2	3 (1.0)	2 (2.0)	1 (1.0)	
	T3	1 (0.3)			1 (1.0)
	T5_RUS1	1 (0.3)		1 (1.0)	
LAM		**4 (1.3)**	**2 (2.0)**	**1 (1.0)**	**1 (1.0)**
	LAM10	1 (0.3)	1 (1.0)		
	LAM9	3 (0.7)	1 (1.0)	1 (1.0)	1 (1.0)
H	H3	**3 (1.0)**	**3 (3.1)**		
Zero		**1 (0.3)**		**1 (1.0)**	
U		**24 (8.0)**	**6 (6.1)**	**8 (8.0)**	**10 (10.0)**
Total		**298 (100)**	**98 (100)**	**100 (100)**	**100 (100)**

The bold numbers are the total numbers of isolates belonging to the *M. tuberculosis* lineages presented on the same lines in the first column, equal to the sum of the numbers of isolates of the sublineages (presented on the second column) under these lineages

The proportions of Beijing isolates were higher in the North (70.4 %) and the South (68 %) than in the Centre (28 %) (OR = 1.7 [95 % CI: 1.4–2.0], *p* < 0.0001), whereas the proportion EAI isolates in the North (7.1 %) and the South (17 %) was much lower than in the Centre (59 %) (OR = 0.5 [95 % CI: 0.4–0.6], *p* < 0.0001). The proportions of isolates belonging to lineages other than Beijing and EAI lineages were not significantly different between the regions (Tables 1 and 2). Among EAI isolates, those of EAI4-VNM and EAI5 sub-lineages accounted for the majority. In the North and the South, EAI4-VNM sub-lineage was more prevalent than EAI5 (85.7 % and 64.7 % vs. 14.3 % and 23.5 %, respectively), but in the Centre, this lineage did not predominate compared to EAI5 (46.6 % vs. 51.7 %).

**Table 2:** Comparison of the proportion of *M. tuberculosis* lineages at the regional reference hospitals

Lineage	Region			OR (95 % CI)		OR (95 % CI)	
	North	Centre	South	(North & South) vs. Centre	*P*_a_	North vs. South	*P*_*a*_
	n (%)	n (%)	n (%)				
Beijing	69 (70.4)	28 (28.0)	68 (68.0)	1.7 (1.4–2.0)	<0.0001	1.0 (0.7–1.0)	0.8223
EAI	7 (7.1)	58 (58.0)	17 (17.0)	0.5 (0.4–0.6)	<0.0001	1.6 (1.0–2.6)	0.0419
Others	22 (22.5)	14 (14.0)	15 (15.0)	1.1 (0.9–1.4)	0.2570	0.8 (0.5–1.1)	0.1580

_a_: Logistic regressions, correcting for age and sex of the patients

**Fig. 3:**
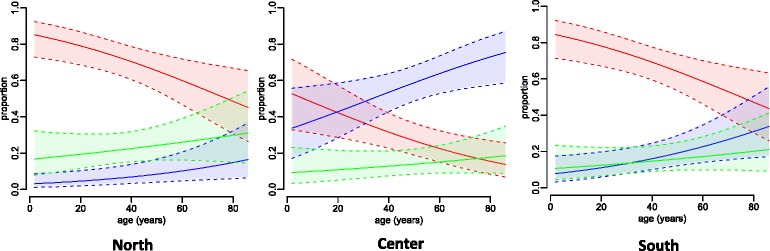
Distribution of *M. tuberculosis* lineages by age at the regional reference hospitals. *Note:* The lines are the logistic model predictions (corrected for patients’ gender) of the proportion of Beijing (*red*), EAI (*blue*) and lineages other than Beijing and EAI (*green*) together with 95 % CI (*shaded area*)

After adjusting for gender and region, the proportion of Beijing isolates remained significantly higher (*p* < 0.01) and the proportion of EAI isolates significantly lower (*p* < 0.05) in patients of younger ages. No age trends were observed for the proportions of isolates belonging to lineages other than Beijing and EAI. Figure 3 shows the distribution of *M. tuberculosis* lineages by age, correcting for gender in the three studied regions. These age differentials in the distribution of lineages did not significantly differ between regions (*p* > 0.9 for Beijing lineage and *p* > 0.2 for EAI lineage).

### Drug resistance of *M. tuberculosis* lineages at the regional reference hospitals

The proportions of isolates that were resistant to RMP, INH, SM and EMB among the *M. tuberculosis* isolates were 24.5 %, 39.6 %, 42.6 % and 16.8 %, respectively. MDR isolates was found in 23.8 % of the total. All but two isolates that were resistant to RMP were also resistant to INH (71 isolates). The proportions of isolates that were resistant to RMP, INH, SM and EMB were significantly lower in the Centre (6 %, 12 %, 14 % and 4 %, respectively) than in the two other regions, which did not significantly differ from each other (49.0 %, 28.6 %, 56.1 % and 23.5 % in the North and 58.0 %, 39.0 %, 58.0 % and 23.0 % in the South) (Tables 3 and 4).

**Table 3:** Drug resistance levels of *M. tuberculosis* lineages at the regional reference hospitals

Resistance	Lineage/ Sub-lineage	Location			
		Total	North	Centre	South
		n _a_ (col.%) _b_	n _a_ (col.%) _b_	n _a_ (col. %) _b_	n _a_ (col.%) _b_
INH	All	**298 (39.6)**	**98 (49.0)**	**100 (12.0)**	**100 (58.0)**
	Beijing	165 (53.9)	69 (58.0)	28 (32.1)	68 (58.8)
	EAI	82 (14.8)	7 (14.3)	58 (3.5)	17 (52.9)
	EAI4_VNM	44 (16.3)	6 (0.0)	27 (3.7)	11 (54.5)
	Others	51 (32.7)	22 (31.8)	14 (7.1)	15 (60.0)
RMP	All	**298 (24.5)**	**98 (28.6)**	**100 (6.0)**	**100 (39.0)**
	Beijing	165 (34.5)	69 (34.8)	28 (21.4)	68 (39.7)
	EAI	82 (9.9)	7 (14.3)	58 (0.0)	17 (41.2)
	EAI4_VNM	44 (11.6)	6 (0.0)	27 (0.0)	11 (45.5)
	Others	51 (15.4)	22 (13.6)	14 (0.0)	15 (33.3)
SM	All	**298 (42.6)**	**98 (56.1)**	**100 (14.0)**	**100 (58.0)**
	Beijing	165 (63.0)	69 (66.7)	28 (42.9)	68 (67.6)
	EAI	82 (6.1)	7 (14.3)	58 (0.0)	17 (23.5)
	EAI4_VNM	44 (7.0)	6 (0.0)	27 (0.0)	11 (27.3)
	Others	51 (34.6)	22 (36.4)	14 (14.3)	15 (53.3)
EMB	All	**298 (16.8)**	**98 (23.5)**	**100 (4.0)**	**100 (23.0)**
	Beijing	165 (25.5)	69 (27.5)	28 (14.3)	68 (27.9)
	EAI	82 (3.7)	7 (14.3)	58 (0.0)	17 (11.7)
	EAI4_VNM	44 (4.6)	6 (0.0)	27 (0.0)	11 (18.1)
	Others	51 (9.6)	22 (13.6)	14 (0.0)	15 (13.3)
MDR	All	**298 (23.8)**	**98 (28.6)**	**100 (6.0)**	**100 (37.0)**
	Beijing	165 (33.3)	68 (34.8)	28 (21.4)	68 (36.7)
	EAI	82 (9.9)	7 (14.3)	58 (0.0)	17 (41.2)
	EAI4_VNM	44 (11.6)	6 (0.0)	27 (0.0)	11 (45.5)
	Others	51 (15.4)	22 (13.6)	14 (0.0)	15 (33.3)

_a_:Total number of isolates

_b_:Proportion of drug resistant isolates

The bold numbers show the total sums and proportions of drug resistance (in bracket) for all isolates, irrespective of their lineages

**Table 4:** Comparison of dug resistance levels of *M. tuberculosis* lineages in the different regions

Resistance	Region _a_			Lineage _b_		
	North	Centre vs. North	South vs. North	Beijing	EAI vs. Beijing	Others vs. Beijing
		OR (95 % CI)	OR (95 % CI)		OR (95 % CI)	OR (95 % CI)
INH	1.0	0.2 (0.1–0.6)	1.6 (0.9–3.0)	1.0	0.2 (0.1–0.6)	0.4 (0.2–0.9)
RMP	1.0	0.2 (0.1–0.6)	1.9 (0.9–3.5)	1.0	0.4 (0.1-0.9)	0.4 (0.2–0.9)
SM	1.0	0.3 (0.1–0.7)	1.3 (0.7–2.5)	1.0	0.1 (0.0–0.2)	0.3 (0.2–0.7)
EMB	1.0	0.2 (0.1–0.7)	1.2 (0.6–2.5)	1.0	0.2 (0.1–0.7)	0.3 (0.1–0.9)
MDR	1.0	0.2 (0.1–0.6)	1.7 (0.9–3.2)	1.0	0.4 (0.2–1.0)	0.4 (0.2–0.9)

_a_:Logistic regressions correcting for *M. tuberculosis* lineage, age and sex of the patients

_b_:Logistic regressions correcting for region, age and sex of the patients

Overall, Beijing isolates were the most commonly resistant ones and EAI isolates were the least commonly resistant ones. This was observed in the North and the Centre, but not in the South where EAI lineage revealed also a high level of drug resistance similar to Beijing lineage (Tables 3 and 4). The EAI4-VNM sub-lineage, which is specific to Vietnam, had drug resistant proportions similar to those of the overall EAI lineage. The lineages other than Beijing and EAI were significantly less resistant than Beijing but more resistant than EAI (Tables 3 and 4).

After adjustment for lineage, region and sex, the proportions of resistance to the first-line drugs were higher in patients of the middle age groups (OR (vs. age quintile I) was 1.4–4.2 for age quintiles II, III, IV, Table 5) and highest for age quintiles III (OR (vs. age quintile I) was 2.6–4.2, Table 5). There was no significant difference between age quintile I and V (OR (vs. age quintile I) was 0.6–1.7 for age quintile V, Table 5). After adjustment for lineage, region and age group, the proportions of MDR and EMB resistance were significantly higher in men than in women (Table 5).

**Table 5:** Association of the drug resistance levels of *M. tuberculosis* with gender and age of the patients by logistic regression analysis

Resistance	Sex (Male vs. female) _b_		Age quintile _a_, _c_				
	OR (95 % CI)	*P*	II vs. I	III vs. I	IV vs. I	V vs. I	*P*
			OR (95 % CI)	OR (95 % CI)	OR (95 % CI)	OR (95 % CI)	
INH	1.5 (0.8–2.8)	0.25	4.1 (1.8–9.6)	4.2 (1.7–10.3)	3.2 (1.4–7.6)	0.8 (0.3–2.2)	0.0002
RMP	2.4 (1.2–4.8)	0.01	2.9 (1.1–7.3)	2.9 ((1.1–7.6)	2.5 (1.0–6.5)	1.1 (0.3–3.6)	0.08
SM	1.5 (0.8–3.0)	0.2	2.0 (0.9–4.6)	2.6 (1.0–6.4)	2.1 (0.9–5.1)	0.6 (0.2–1.6)	0.02
EMB	2.4 (1.1–5.1)	0.02	3.8 (1.3–10.8)	3.8 (1.3–11.4)	1.4 (0.5–4.5)	1.7 (0.5–6.2)	0.04
MDR	2.5 (1.3–4.9)	0.009	2.6 (1.0–6.7)	2.9 (1.1–7.6)	2.3 (0.9–5.9)	1.1 (0.3–3.5)	0.11

_a_: I, II, III, IV and V: age quintile, the cut-points for age quintiles are 2 (min), 28, 37, 46, 57 and 86 (max)

_b_: Correcting for region, lineage and age

_c_: Correcting for region, lineage and sex

## Discussion

### The *M. tuberculosis* lineages from reference hospitals across Vietnam

Our study showed that the most predominant *M. tuberculosis* lineage was Beijing, especially in the North and the South. Compared with a study conducted in isolates exhaustively acquired in the same reference hospitals in the North and the South during 1998 and the first quarter of 1999, which showed Beijing strains were emerging [11], the proportion of Beijing isolates had increased (from 54.0 % in previous study to 68.5 % in this study). As suggested in our previous study, Beijing strains would have been imported through international exchanges and migration and spread within urban areas and from urban to rural areas [10]. The increase in the proportion of Beijing isolates during the 10 years that separate the 1998–1999 study and the current one was thus expected.

The difference in the proportions of Beijing isolates in the Centre compared with the North and the South can be explained by the differences in regional characteristics. In particular, the reference hospitals in the North and the South are located in the two most major and largest cities of Vietnam, whereas the reference hospital in the Centre is located in Hue city which is a small city and more isolated in terms of international exchanges and migration. This may reflect the rural and urban disparities in the genetic spectrum of *M. tuberculosis* isolates as previously documented [10].

Our spoligotyping results showed that the *M. tuberculosis* lineages were globally the same in the three regional hospitals, apart from 1 or 2 isolates. The *M. tuberculosis* sample in this study was genetically less diverse than the sample collected at low-level hospitals in the Northern plain of Vietnam in our previous study [10]. In particular, the MANU and X lineages, which accounted for 2.2 % and 0.8 % respectively in the previous study, were absent from the isolates collected from the regional hospitals.

TB that occurs in young patients is likely to be attributable to recent transmission whereas in older patients it is more likely to be attributable to reactivation of latent TB that was acquired when the person was young [27]. Hence, the observed association of *M. tuberculosis* lineage distribution with patients’ ages (that is, a higher proportion of Beijing isolates and a lower proportion of EAI isolates in younger patients), which is consistent with previous studies in Vietnam [10, 28], suggests a progressive replacement of EAI strains with Beijing strains in all studied regions. As Beijing isolates were less prevalent and EAI isolates were more predominant in the Centre compared with the North and the South, this replacement seems to be less intensive in the Centre. It is noteworthy that in the regions with more intensive replacement, the proportions of EAI5 isolates out of the total EAI isolates were lower than in the region with less intensive replacement (14.3 % in the North and 23.5 % in the South vs. 51.7 % in the Centre). Conversely, the proportions of EAI4-VNM isolates out of the total EAI isolates were higher in the regions with more intensive replacement (85.7 % in the North and 64.7 % in the South vs. 46.6 % in the Centre). This suggests that the EAI5 sub-lineage was more prone to be replaced compared with the EAI4-VNM sub-lineage. The replacement of locally adapted EAI strains with Beijing strains as seen in this and previous studies suggests, as expected, a particularly higher fitness of Beijing strains compared to EAI strains in Vietnam.

### The association of *M. tuberculosis* lineage and anti-tuberculosis drug resistance in reference hospitals across Vietnam

The overall proportions of resistance to the four first-line anti-tuberculosis drugs of the studied *M. tuberculosis* isolates were the highest for SM (42.6 %) and the lowest for EMB (16.8 %). MDR isolates accounted for 23.8 % of the total. The levels of drug resistance observed in this study were similar to the levels of drug resistance in patients who were being treated for failure or recurrent TB during the National Drug Resistance survey, conducted in 2011 [29]. This is consistent with the strategic role of reference hospitals in the Vietnam National TB Program.

As the Vietnam National TB program has been using GeneXpert for MDR-TB case detection, the high proportion (97.3 %) of INH resistant isolates that are also resistant to RMP in our study suggests that this strategy is reliable.

Our analysis shows that the prevalence of drug resistance was lower in the Centre than in the rest of the country and was not significantly different between the North and the South. This is explained by the lower proportion of Beijing isolates and the higher proportion of EAI isolates in the Centre than the rest of the country and the finding that Beijing isolates are more likely to be drug-resistant than EAI isolates. This finding suggests that the ongoing replacement of EAI lineage by Beijing lineage in Vietnam also means a replacement of the most drug-susceptible strains by highly drug-resistant strains. Therefore, not withstanding acquired resistance caused by TB treatment, a higher rate of drug resistant TB can be foreseen in the near future. The empirical evidence to justify this concern is the increased rate of MDR-TB, as shown in consecutive National Drug Resistance surveys, from 2.7 % to 4 % among new cases and from 19 % to 23.3 % in retreatment cases between 2006 and 2011 [21, 22].

In the South, both the Beijing lineage and the EAI lineage were associated with a high likelihood of being drug-resistant. This latter lineage was least likely to be associated with drug resistance in the North and the Centre. The EAI4-VNM sub-lineage was reported to be the predominant one (50.7 %) in a study conducted in 3 rural districts in the South of Vietnam [28], with a prevalence almost twice as high as the one in rural districts in the North of Vietnam [10]. In this study EAI/ EAI4-VNM isolates were found to have very different levels of drug resistance in the three regions. This lineage/sub-lineage was among the most drug-resistant lineages in the South but was the most sensitive in the North and the Centre. This implies that these strains are also epidemiologically important and need to be monitored, and there would be a higher antibiotic pressure in the South compared to the North and the Center.

The observation association between male-to-female ratio and patients’ age for the studied *M. tuberculosis* isolates was similar to the finding of the National TB prevalence survey of Vietnam conducted during 2006–2007. This survey also showed that the male-to-female ratio for prevalent TB was higher in patients of working ages (25 to 64 years) and lower in the younger and older patients [21]. The high overall male-to-female ratio in this study is also consistent with findings of the prevalence survey and other previous studies in which the male-to-female ratio was between 2.4–5.1 [10, 14, 21]. These findings demonstrate that the observed gender difference in this study population is not an artefact of sample collection from the reference hospitals or related to gender differences in access to diagnosis and treatment, but it is actually a feature of epidemiology of TB in Vietnam in general.

For all the tested drugs, the proportions of drug-resistant isolates were higher in patients of middle age groups (28–57 years old), highest for patients of 37–46 years old and lower in patients of younger and older ages. Furthermore, the proportions of MDR and EMB resistant strains were significantly higher in men compared to women. These findings are different from those of a study conducted in Georgia among hospitalized TB patients, which failed to show any association between drug resistance and age [30]. This study also showed that female patients were more at risk of having MDR-TB than male patients. These differences suggest that control strategies should be adapted for different settings. Studies in developing countries showed that male TB patients tend to have poorer treatment compliance than female ones due to neglected treatment, alcoholism and fear of losing jobs, especially the ones of the middle age groups [31, 32]. This problem could lead to higher level of drug resistance among male TB patients. In order to achieve better control over the emergence of MDR-TB in Vietnam, it is important to focus on improving treatment compliance among male patients of working ages.

## Conclusions

This first study on the molecular epidemiology of TB in TB reference hospitals across regions of Vietnam shows that Beijing and EAI lineages were the most epidemiologically important ones, not only because they are the most predominant lineages/sub-lineages but also because they are the most likely to be drug-resistant lineages in one (in the South for EAI lineage) or all regions of Vietnam (for Beijing lineage). The replacement of EAI strains by Beijing strains, especially in the North and in the Centre can be expected to lead to a higher prevalence of drug resistant TB in Vietnam in the near future. Male patients of working ages should be particularly targeted in order to prevent the emergence of drug resistant TB.

## Abbreviations

DST, drug susceptibility testing; EMB, ethambutol; INH, isoniazid; MDR, multi-drug resistant; RMP, Rifampicin; SM, streptomycin; TB, tuberculosis; XDR, extensively drug-resistant

## Additional file

Additional file 1:
*M. tuberculosis* isolates at the regional reference hospitals of Vietnam. (XLS 333 kb)
